# Stakeholders’ perspectives on implementing and integrating patient-reported outcome measures (PROMs) in health systems - insights from Alberta, Canada

**DOI:** 10.1186/s41687-025-00887-0

**Published:** 2025-06-05

**Authors:** Fatima Al Sayah, Hilary Short, Arto Ohinmaa, Stafford Dean, Brenda Hubley, Markus Lahtinen, Jeffrey A. Johnson

**Affiliations:** 1 Alberta PROMs and EQ-5D Research and Support Unit (APERSU), School of Public Health, University of Alberta, Edmonton, Alberta Canada; 2 Alberta Health Services, Calgary, Alberta Canada; 3 Cancer Care Alberta, Edmonton, Alberta Canada; 4 Health System Analytics, Health Quality Council of Alberta, Calgary, Alberta Canada

**Keywords:** Patient-reported outcome measures, Routine outcome measurement, Health system, Canada

## Abstract

**Background:**

Patient-reported outcome measures (PROMs) are valuable tools for evaluating outcomes of healthcare interventions and have been increasingly used in health systems around the world. While PROMs adoption has grown globally, variations exist in their use across the health system. This stakeholder engagement and consultation activity aims to understand factors impacting PROMs adoption, implementation, and utilization, and identify strategies for enhancing utilization of PROMs data within the healthcare system in Alberta, Canada.

**Methodology:**

Key stakeholders from various roles were interviewed, including PROMs programs, health system improvement, data analytics, clinical practice, evaluation, health economics, and research, from diverse clinical areas, including cancer, primary care, epilepsy, rehabilitation, arthroplasty, cardiovascular surgery, and rheumatology. Interviews were recorded, transcribed and analyzed using thematic analysis. Results were synthesized using the Consolidated Framework for Implementation Research (CFIR) and Diffusion of Innovation Framework (DOI).

**Results:**

A total of 25 interviews, lasting between 45 and 60 minutes, were completed. Factors impacting the adoption, implementation and utilization of PROMs within the Alberta healthcare system included: (1) Intervention characteristics: shared understanding of PROMs, challenges in capturing relevant patient outcomes, standardization and integration of PROMs, modalities of data collection, making PROMs actionable at the patient-clinician level, and interpreting PROMs data; (2) Inner setting: Cultural shift towards patient-centered care, change management and clinician mindset, organizational commitment and support, integration into broader measurement frameworks, access to PROMs data, potential for replication and adaptation, and importance of incentives and requirements; (3) Outer setting: Resource constraints, policy and systemic challenges, focus on value-based care, and responsible use of PROMs data; (4) Characteristics of individuals: Expertise and understanding of PROMs, and stakeholder engagement and education; and (5) Process: Balancing bottom-up and top-down approaches, workflow integration, patient engagement, and continuous evaluation and quality improvement.

**Conclusions:**

The study highlights factors influencing PROMs adoption in Alberta, including the need for a unified understanding, workflow integration, and electronic data use. Key strategies involve fostering patient-centered care, ensuring organizational support, addressing resource and policy issues, and providing targeted education. Engaging early adopters and offering incentives can improve PROMs integration and patient outcomes.

**Supplementary Information:**

The online version contains supplementary material available at 10.1186/s41687-025-00887-0.

## Background

Patient-reported outcome measures (PROMs) are direct reports from patients on how they feel about their own health or an aspect of it, without interpretation from anyone else [1]. PROMs can be generic or disease-specific, and they can assess health-related quality of life, well-being, functional status, or symptoms burden. PROMs play an increasingly significant role in healthcare systems by providing valuable tools to evaluate patients’ experiences and treatment outcomes [2–9]. Unlike traditional clinical measures, PROMs capture patients’ perspectives on various aspects of their health, enabling a more comprehensive assessment of the impact and value of healthcare interventions on patients’ health. By systematically measuring and monitoring PROMs, healthcare organizations, such as hospitals, primary care centers, or speciality clinics, can make data-driven decisions, identify patterns in care, and benchmark performance against established standards [5, 10]. PROMs data can serve as a foundation for quality improvement initiatives, evidence-based decision-making, and ultimately enhance the quality of care.

The adoption and use of PROMs in healthcare systems, as seen globally [11, 12], has gained significant momentum in the province of Alberta, Canada. Over the past decade, healthcare organizations across the province have embraced the implementation of PROMs, leading to their widespread application in a variety of clinical settings [13–20]. In areas where implementation has been successful, PROMs have provided invaluable insights into assessing the impact of healthcare interventions on patient outcomes, fostering a deeper understanding of the value of these interventions from the patients’ perspective. Despite this progress, the routine PROMs collection and use of PROMs varies widely across the Alberta healthcare system. While efforts have primarily focused on optimizing data collection, there has been a relatively less focus on leveraging PROMs data for various applications within the system, such as informing clinical decision-making, guiding healthcare planning, supporting quality improvement initiatives, and facilitating resource allocation decisions. Recognizing this crucial gap, a comprehensive multi-level framework has been developed to guide and optimize the use of PROMs data across the micro (patient-clinician), meso (organization), and macro (system) level [21]. These factors encompass individual patients’ and clinicians’ understanding and attitudes toward PROMs, administrative support for their implementation—including the availability of resources—and system-level considerations such as regulations, guidelines, and policies governing the collection and use of PROMs data for purposes like quality improvement and benchmarking.

Extensive research has shed light on various factors that affect the implementation of PROMs in specific clinical settings and the utilization of PROMs data [3, 6, 22–25]. However, these factors are intricately tied to individual healthcare systems, their unique characteristics, and the local context. In the province of Alberta, there has been substantial dedication to integrating PROMs into the healthcare system, as evidenced by significant efforts to establish standardized PROMs capture through patient information systems [13]. However, to effectively leverage PROMs data, it is essential to devise a set of strategies and best practices that align with Alberta’s healthcare system and its particular characteristics. This initiative was designed to gain a deeper understanding of the local contextual factors that influence the adoption, implementation and utilization of PROMs.

This project seeks to foster meaningful engagement and gather invaluable insights from key stakeholders involved in patient-centered outcome measurement, with a specific focus on PROMs, within the Alberta healthcare system. The main goals were to:

- Understand stakeholders’ views on the adoption and use of PROMs, assessing their experiences to identify strengths, challenges, and opportunities for integrating PROMs into the healthcare system.
- Identify strategies and priority areas to enhance PROMs use in the health system.

## Methods

### Context: Alberta’s healthcare system

Alberta’s healthcare system is publicly funded, serving around 4.8 million residents. The provincial government, through the ministry Alberta Health, governs the healthcare system, which is delivered by a single regional health authority, Alberta Health Services (AHS) and primary care networks. Patient-centered care is emphasized in the Alberta healthcare system, with a focus on engaging patients in decision-making processes, respecting their values and preferences, and promoting shared decision-making. Alberta has increasingly invested in the implementation of PROMs within the healthcare system, which led to the establishment of the “Alberta PROMs and EQ-5D Research and Support Unit” (APERSU); a center of excellence to support the use of PROMs, guiding and supporting the adoption and implementation of these measures within the health system.

This project was conducted as a professional engagement activity, guided by principles of stakeholder involvement, and as such, formal ethics approval was not required. However, the study adhered to the ethical guidelines outlined in the Canadian Tri-Council Policy Statement: Ethical Conduct for Research Involving Humans [26]. Participants were informed about the study’s purpose and provided oral consent to participate. They also consented to have their interviews recorded and for their data to be shared in an aggregated, anonymized form.

### Stakeholder selection and recruitment

Participants for this initiative were selected through the APERSU network, which includes a diverse group of collaborators and stakeholders actively engaged in PROMs-related work across Alberta. We used a purposive sampling approach to ensure that a broad range of perspectives from various PROMs end-users and stakeholders were represented in the project, including clinicians, administrators, and decision-makers. Additionally, key stakeholders from major health organizations across the province were included, such as Alberta Health (the Ministry of Health), healthcare delivery institutions like Alberta Health Services, health-quality assurance bodies such as the Health Quality Council of Alberta, and specialty organizations like Cancer Care Alberta. The perspectives of these stakeholders on the adoption and use of PROMs—and strategies for enhancing their utilization—were expected to differ across these settings, so it was essential to ensure comprehensive representation. To further strengthen the diversity of perspectives, recommendations from the participants were sought to identify other key stakeholders to be interviewed from their area of work or related areas. This inclusive approach ensured a comprehensive representation of stakeholders and facilitated connections with individuals who hold significant influence in shaping PROMs-related initiatives in Alberta.

### Stakeholder engagement

This stakeholder engagement and consultation activity has been guided by a comprehensive framework known as the “Engagement Cycle” [27]. The Engagement Cycle encompasses five distinct levels of stakeholder engagement: inform, consult, involve, collaborate, and empower. Each level represents a progressive degree of stakeholder involvement, ranging from simply providing information to actively empowering stakeholders in decision-making processes. Throughout this activity, our team has diligently followed the principles of the Engagement Cycle, ensuring that stakeholders were informed, consulted, and involved in the implementation of PROMs in Alberta.

### Data collection

To gather insights from PROMs stakeholders in Alberta, we conducted individual key informant interviews, which were held virtually using a semi-structured interview guide (Appendix 1). The interview guide was developed by FAS, with all team members providing input on it. The interviews took place between March and June 2023 and were conducted by FAS and HS. Some participants were existing collaborators of the study team, with prior working relationships on PROMs initiatives in the province. These established connections played a crucial role in facilitating engagement and enriching the conversations during the interviews. All interviews were recorded, transcribed verbatim, and reviewed for accuracy. The transcriptions served as the primary data source for analysis. The interviews covered a wide range of topics related to the adoption and use of PROMs data in healthcare settings, including healthcare service planning and evaluation, quality improvement, healthcare system performance assessment, the setting of provincial health priorities, and the exploration of outcome-based payment models.

### Data analysis

We used a thematic analysis approach to analyze the interview data, which involved identifying emerging concepts, refining code definitions, and developing overarching themes. This process was conducted by two study team members who conducted the interviews, using Microsoft Word and Excel. Any discrepancies in coding were resolved through discussion and consensus between the two coders. The results were regularly reported and discussed with the other team members. We mapped the identified themes to the Consolidated Framework for Implementation Research (CFIR). CFIR is a comprehensive framework designed to systematically assess and understand the factors that influence the implementation of complex interventions [28]. It organizes factors into five main domains, including intervention characteristics, outer setting, inner setting, characteristics of individuals, and process. The CFIR framework has been widely utilized in implementation research related to PROMs, and its use in this project facilitates comparability with research from other regions. Additionally, we used the Diffusion of Innovation (DOI) to synthesize results on strategies to improve adoption and use of PROMs in health systems [29]. The DOI framework, with its focus on how innovations spread within a system, was particularly useful for identifying strategies to overcome barriers and accelerate the uptake of PROMs in diverse healthcare contexts.

Throughout data collection, we continuously synthesized and analyzed the data to identify key themes. After the first 18 interviews, common themes began to emerge pointing to data saturation, which we validated through the subsequent seven interviews. During these interviews, respondents were specifically asked to provide feedback on the identified themes, enhancing the credibility and reliability of our findings.

Finally, we presented the findings at a town hall discussion held during the APERSU annual meeting in October 2023. This meeting, attended by over 70 PROMs end-users from various clinical settings, provided an opportunity for stakeholders to discuss and validate the findings. No changes or revisions to the identified themes were proposed in this townhall discussion.

## Results

A total of 36 key stakeholders were identified and invited to participate in the interviews. Of these, 11 individuals either did not respond (n = 7), declined and suggested alternative participants (n = 2), or were unavailable for the interview (n = 2). Ultimately, 25 stakeholders (response rate 69.4%) from various healthcare settings and organizations in Alberta participated in the interviews. These included Alberta Health (n = 4), Alberta Health Services (AHS, n = 10), Primary Care Networks (n = 4), and other institutions such as the Health Quality Council of Alberta (n = 2), Institute of Health Economics (n = 1), University of Alberta (n = 2), University of Calgary (n = 4), Cancer Care Alberta (n = 3), and the Alberta Bone and Joint Health Institute (n = 1). Participants held a range of roles, including PROMs program leads (n = 10), health system administrators (n = 3), data analytics leads (n = 3), clinicians (n = 7), evaluators (n = 2), health economists (n = 2), and academic researchers (n = 3). Some participants were affiliated with multiple organizations and held various roles. The sample also included representatives from diverse clinical areas, such as cancer, primary care, epilepsy, rehabilitation, hip and knee arthroplasty, cardiovascular surgery, and rheumatology. Notably, six stakeholders were directly involved in using the EQ-5D-5L in population health surveys across Alberta. Interviews lasted between 45 and 60 minutes, with an average duration of 56.4 minutes.

### Factors impacting PROMs adoption and implementation

**Fig. 1:**
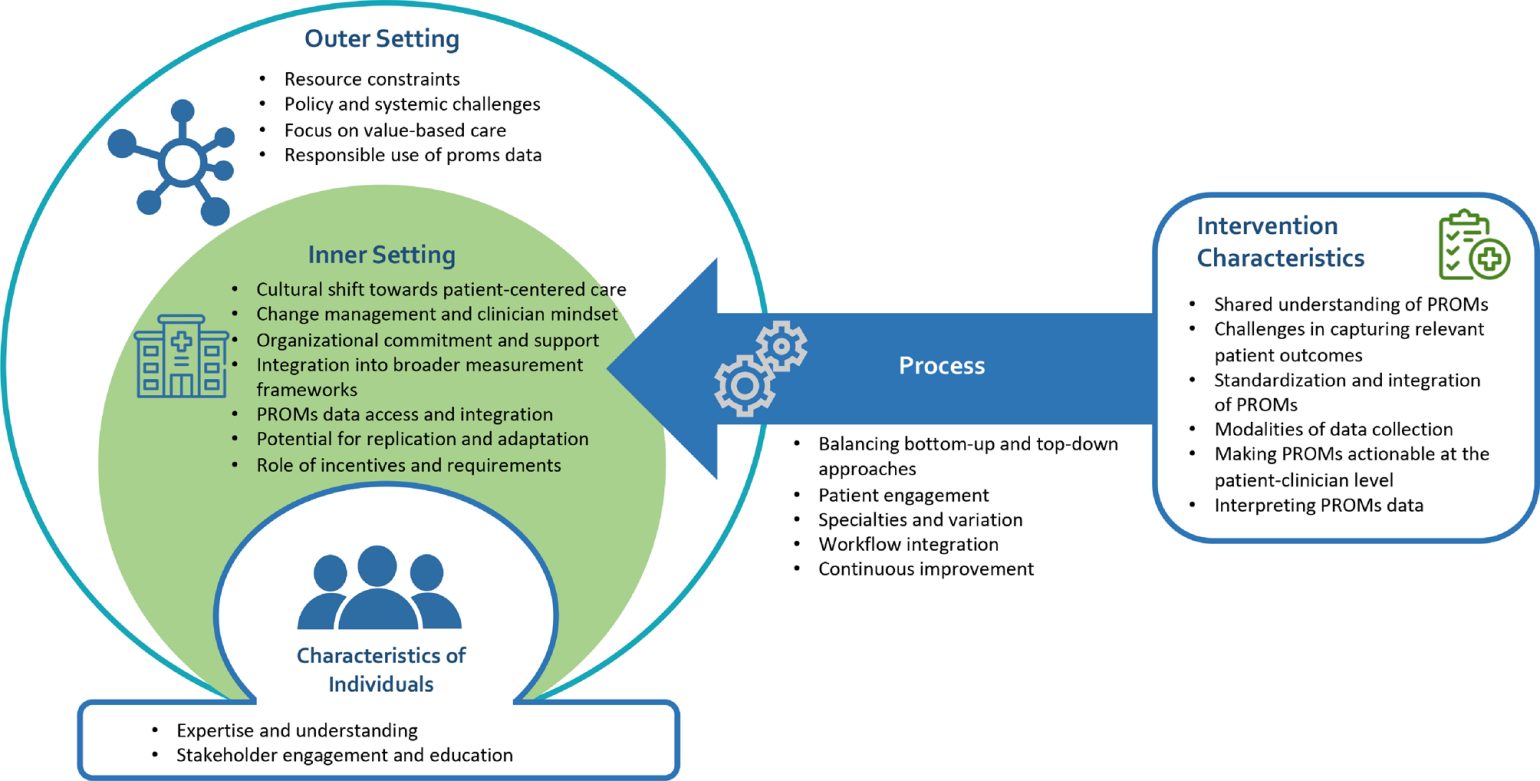
Factors affecting PROMs adoption and implementation in Alberta based on the CFIR framework

The analysis of stakeholder perspectives on the adoption and use of PROMs, aligned with the CFIR framework, reveals several key themes across the domains of Intervention Characteristics, Inner Setting, Outer Setting, Characteristics of Individuals, and Process (Fig. 1). Direct quotes from participants supporting each of themes and sub-themes are presented in Table 1.

**Table 1:** Quotes from participants on each identified theme in the CFIR Framework

CFIR Domain	Identified themes	Quotes from participants
Intervention Characteristics	Shared understanding about PROMs	*“Coming to a common and a shared understanding of what PROMs are … looking really at what those users or what our clinicians believe and have intended or think what PROMs mean in their mind.” (P02, Administrator)* *“I don’t think our frontline providers understand the accountability that our PCNs are under and the requirements, and it’s not for the lack of communication. I think people are scared because they’re going to be held to a higher account as well.” (P17, Administrator)* *“Raising [PROMs] awareness [with patients] is very important. This is a way for you to tell me how you feel. It’s a way for you to tell me to express what are the important things for you. It’s a way for you to tell me your story and what really matters to you because people are not really used to it.” (P27, Researcher)*
	Challenges in capturing relevant patient outcomes	*“I am involved with trying to find measures that would be useful for chronic pain … So, the working group has decided not to go with the EQ-5D only because even though pain is one of the dimensions. It’s still not as clear, like in terms of the different components of pain that we want. It’s too generic. So it’s not giving us exactly what we want, like functional, emotional, physical pain.” (P13, Evaluator)* *“[PROMs] have to be population specific. [PROMs] has to be normalised or whatever for whatever population you’re dealing with … I think it would be a hard sell to get [physicians] who are not invested [in outcome data] to measure EQ-5D. They wouldn’t see the value, right? Something like a PHQ is very specific and it’s easy to measure and it helps us understand if somebody is actually changing over time.” (P19, Physician)* *“I think some [PROMs] questions that get asked are really generic … Do they really capture the same thing in all populations or just the general population? Would I be able to use this question to get the same answer if I asked the East Indian community or the indigenous community? That’s the part where I just am not sure.” (P25, Evaluator)* *“One area I’ve been doing this is looking at differentiation, functioning and response shift. Looking at heterogeneity and how people respond and interpret questions. Some of the work I’m doing looking at large populations and trying to see differences in our people’s health report, differences in terms of effect sizes and improvement and stuff like that. And that worries me that at population level, are we going to end up comparing apples and oranges, especially quality of life, if we’re using these PROMs at national level or at large populations where many things can play a role in that?” (P18, Researcher)*
	Standardization and integration of PROMs	*“When we are using [PROMs], as a pre and post measure, comparing a change in quality of life post-rehab, we’re not using it in isolation … We try to say [PROMs are] one piece of the puzzle when looking at service improvement, that it provides important information, and it’s really the only, or one of the few bits of data that’s collected directly from the patient’s perspective.” (P04, Administrator)* *“And the thing is, it’s hard to standardize across the system because different organizations are going to be at such different levels and so it’s going to look good in one area, look bad in the other, but it may not necessarily be the outcomes or like the services that are being, that are inadequate.” (P21, Evaluator)* *“I think that there’s real value in having [PROMs] information if we can find the right level at which to present it and find ways in which people could use it. It would have to be some sort of composite of things so some kind of patient experience, some kind of outcomes related to clinical areas, some kind of information on wait times. Not stand-alone outcome on its own.” (P05, Researcher)*
	Modalities of data collection	*“We’ve done paper, we’ve done electronic, if it can be done automatically, that it’s on an iPad or with a link sent in advance that it doesn’t take up clinical time we found that’s a bit of the sweet spot.” (P07, Administrator)* *“I’m not sure exactly even how [PROMs] are often collected … But there needs to be some [electronic] mechanism in place. So it’s not just a one off in a certain Primary Care Network or something, but it’s more Alberta across the entire province. The scope has to be Alberta wide. It has to be.” (P17, Evaluator)*
	Making PROMs actionable at the patient-clinician level	*“I think a lot of people need to be shown how it can benefit them … how does it improve the way I deliver care to my patients? Administrators will want to see how it will improve the service that we’re offering or how it will make my hospital more efficient or more effective.” (P05, Researcher)* *“For an individual patient, [PROMs] help me know what I’m walking into in the room for and to prepare a little bit. If patients have many, many symptoms, I will intentionally sort of acknowledge the difficulty that they’re having clearly by having so many high symptoms, but use it as a way to help prioritize. What’s the most important thing for you today? Let’s make sure we deal with that … When you have data over time, it’s really helpful. So something’s up. Is this new up or has it always been up? Then the approach is very different when you go into the room. I think it helps you catch things that you might have not necessarily asked about otherwise.” (P01, Physician)* *“And then we use [PROMs reports] to drill into the individual trends to actually see the details of what is making this person be flagged high symptom complexity. And that’s where the care actually is and centred on that.” (P08, Administrator)* *“Whereas [clinicians] haven’t really been as well trained on softer outcomes like quality of life and that sort of thing, I think developing algorithms or systems, that kind of protocolize, what to do under these circumstances might also increase engagement because we know it matters to patients.” (P22, Physician-Researcher)*
	Interpreting PROMs data	*“That’s exactly what we hear back from docs, ‘well, where’s the utility in [PROMs data]?’ Because we will compare your patient panel to the aggregate of the EQ-5D, we have those kinds of norms. And you can eyeball whether your panel is generally healthier or less healthy than the norm.” (P14, Evaluator)* *“So you’ll remember that symptom complexity was something we designed in our programme because we needed a summary score that would give the clinician that quick view of how many symptoms the patient was experiencing and the severity of the symptoms that they were having, and really categorise patients encounters into kind of high, medium, low symptom complexity.” (P08, Administrator)* *“Use PROMs, but also be cautious that people are different in the way they are responding … The way I’ve always kind of structured [PROMs interpretation] is to bring the principles of EDI equity, diversity, and inclusion, and to apply an EDI lens to make sure that we’re not painting everybody with the same brush, right?” (P18, Researcher)* *“So I think using [PROMs] as a measure of change is as an important factor. We have looked at how the results change and vary across different types of services, different zones, as well as different client populations. We are finding, using the data to look at trends along services as well as if there are differences with specific health condition populations.” (P04, Administrator)*
Outer Setting	Resource constraints (financial, human, time, technological)	*“There are always the reasons why not. And time was generally the big one. We don’t have time … So it really required that to set the expectations of building workflows, building processes that support and enable it” (P02, Administrator)* *“[PROMs implementation] certainly does take time … we have different operational leaders coming and going to engage the staff is that you have to do in the training efforts and all that sort of fun stuff just to sustain it, maintain it and keep evolving, and remain relevant.” (P09, Administrator)* *“Clinicians are super busy as it is. Access is a big challenge right now. Just seeing your patients, let alone improving the outcomes for them and all these sorts of things is a big challenge.” (P15, Administrator)* *“I wonder if maybe [lack of PROMs uptake] is just a matter of competing priorities, and perhaps PROMs and health related quality of life is a bit more of a big box type item.” (P24, Evaluator)* *“We don’t have enough nurses to keep up like we’re behind and we lack that support. So what’s happened is [PROMs are] being pushed aside and it’s just how many patients can we see, get them on their biologics, fill in their insurance stuff. And so the first thing to go is all this additional [PROMs] stuff.” (P26, Physician-Researcher)*
	Policy and systemic challenges	*“I truly think [PROMs uptake is] dependent on overcoming some traditional working cells and governance structures in the province.” (P12, Researcher)* *“I think one of our big challenges is this healthcare system. The leaders in government don’t have a vision, don’t have a strategy, don’t have anything. It’s a political monster that’s totally being managed by a political engine and telling us who to work with and what to do.” (P15, Administrator)*
	Focus on value-based care	*“A lot of it comes down to money as opposed to outcomes. And I think that probably what we need to focus on is finding cost efficient ways of improving PROMs data.” (P22, Physician-Researcher)* *“The end goal is, what does [PROMs implementation] cost? And what are we getting for it? … [PROMs are] a value to the patient and be able to demonstrate that, which we don’t do.” (P23, Researcher)*
	Responsible use of PROMs data	*“So, going back to do Albertans know the data that we collect and how they can access that data and within that the PROMs and the PREMs and how that might influence or inform the development of healthcare initiatives in the province. They don’t know about that.” (P11, Researcher)* *“I think it’s super important to report back to [patients]. I always think it might even be of a bit of an ethical question as well. If a patient is giving [their time] to fill out these PROMs, we kind of owe it to them to report it back.” (P24, Evaluator)*
Inner Setting	Cultural shift towards patient-centered care	*“We’re still in a system, the volume-driven, administrative data-driven, so we’re being held to account for wait times and flow again. Nobody’s talking about outcomes.” (P15, Administrator)* *“We talk about the importance of patient-oriented research, the importance of listening to [patients] and hearing what they have to say and this is being implemented. But the problem is where does this go? And I think that’s where the major issue is. A lot of people would say, ‘we’ve been involved in some projects where we’re going to collect information from patients’ … [But] are you going to follow up on this? No. Are you going to act on it? No. Is this something that you probably will use when the patient comes in? Probably no. Because it’s not part of your practise, it’s not part of what you do.” (P27, Researcher)* *“You’ll know that you get an appointment time, and it’s kind of given to you, and you go and turn up and wait for 40 minutes and then be seen for five minutes and then go to another room and wait for 30 minutes. And that’s not a patient centred system. That’s a system designed to flow patients through by volume, not by experience. So, I think we’re a long way from a patient centred system, but I think there are ways for PROMs, and particularly in patient experience measures that drive it.” (P05, Researcher)*
	Change management and clinician mindset	*“Because what you’re talking about is actually changing how every clinician and every patient interaction occurs, and that takes clinicians changing their behavior and believing possibly that the patient has something to tell them that they don’t already know.” (P08, Administrator)* *“As a system we’ve just recognized patient centeredness. That concept’s six years old, at a minimum … I think you’re struggling with a system that has a traditional culture of medicine and health professional training where you have the clinician as the technical competent expert who defines health.” (P12, Researcher)* *“I’ve come to the conclusion that it takes time to change a physician’s mind … I’ve come to realise that different groups have different exposures to PROMs and what they’re comfortable with as well.” (P18, Researcher)* *“Change management, I think that’s key … I think we need to have some really strong change management principles in place to help move [PROMs] along so we also don’t lose sight of where we are.” (P20, Evaluator)* *“We need to do more capacity building around PROMs and about outcome data in general. Not just PROMs being one element of it, the culture of how we do care. We’re quite focused on hard outcomes. We’re quite focused on certain ways of how we do care, how we do medicine.” (P22, Physician-Researcher)*
	Organizational commitment and support	*“I think the biggest thing to help was actually direction in many ways. Setting out the expectation versus the option … It is an organizational commitment that is absolutely critical.” (P02, Administrator)* *“But I think because of how AHS is organized, that [PROMs are] coming down through the operational part of the organization. Implementation is a bit stronger. And I think the nurses do use [PROMs] and just see it as part of what their roles and responsibilities are with patients.” (P01, Physician)*
	Integration into broader measurement frameworks	*“Frameworks that we were looking at as being part of AHS and in using the Quadruple Aim, and looking at the patient experience, the patient outcome, the clinician experience and the value or return on investment value for money, components of our services … So we really wanted to make sure that [PROMs] were a core component of our rehabilitation model of care that we’ve started to implement the last few years in the system in community and outpatient and specialized rehabilitation areas.” (P09, Administrator)* *“We have been implementing the EQ-5D-5L as part of a large initiative with Alberta Health Services regarding Community Rehabilitation implementation and or model of care implementation and redesign. It was one piece of a larger implementation to really help advance evidence informed, patient-centered, equitable, accessible Rehabilitation Services, which is a big ambition, but it was one piece of that work.” (P04, Administrator)* *“So my vision statement is high, reliable, integrated clinical care processes that deliver excellent outcomes at acceptable experiences and affordable cost. That’s the Triple Aim [framework] … I want to trace patients, and I want to know that when they go through those paths, that path delivers experiences, it delivers outcomes both clinical and patient-reported, and it costs something.” (P15, Administrator)*
	PROMs data access and integration	*“But [as a] researcher, it’s really hard because I don’t have [PROMs data] access so, actually, Connect Care is making more of a barrier for me to use those data at a higher level actually. It’s the flip side because I don’t have access to Connect Care. I’m not AHS, so I don’t have access to it anymore. I actually have less access than I used to.” (P03, Researcher)* *“The focus and again, I’m a bit biased because this is my area of interest, really should be [PROMs data] linkage. [PROMs data] really needs to be able to link to both causality and eventual outcomes. And the problem right now is that even within EPIC, I feel that it exists somewhat in isolation. It’s hard to export a research ready data set from there that can allow us to explore more detail about what the driver problems are and then what the outcomes of those problems are.” (P22, Physician-Researcher)* *“If I could wave a magic wand, ideally I wish there was better integration around health information shared between AHS and PCNs, because that’s the heart of [the issues]” (P06, Administrator)* *“Having direct information transfer under the HIA, it is appropriate within the confines of that patient’s care to share that information, including if, that could include PROM data. We have an issue where we have private EMR vendors, at least in the primary healthcare system, that we have physicians that have purchased their own EMR as their own business software. Some have less or more compatibility, and we’re trying to get around it in a number of ways, but I think one of the biggest challenges or barriers is just around the sharing of information.” (P07, Administrator)*
	Potential for replication and adaptation	*“But finding those examples of where it’s actually making a difference. You can take it to physicians and say ‘actually, in cancer care, they changed how they triage or they changed the different types of appointments being offered because they saw that it made a difference using PROMs’. Take that and say to some other service ‘hey, you can do this too’.” (P05, Researcher)* *“Edmonton O’daymin [Primary Care Network], which is I think, probably one of the more established PCNs in the province. And I recall their presentation, everything they have is pretty much automated. They have very sophisticated evaluation processes in place and I think showing that kind of what that gold standard can be.” (P21, Evaluator)* *“I think [PROMs uptake] is really around having champions at the right tables to encourage the use of it.” (P04, Administrator)*
	Importance of incentives and requirements	*“If there’s a specific incentive or requirement in place in the system that’s going to trigger [PROMs use] … In the end I think that’s what’s really going to trigger it because you get everybody’s attention if it’s right and if it’s linked to payment. I hate to say it, but it changes the way people behave.” (P02, Researcher)* *“I think at hospital level, you could use [PROMs] to drive some quality rewards system and say ‘okay, if you want, there’s money on the table. If your patients report good experiences through these PREMs or PROMs or whatever, there’s money on the table for that.’ That’s an option.” (P05, Researcher)*
Characteristics of Individuals	Expertise and understanding of PROMs	*“I think there’s probably a gap, a skills gap as well, within services on how to use that kind of data”.* *“I think it’s challenging to use [PROMs for] individual change … the clinicians are afraid that it’ll be used to measure the effectiveness of their treatment, when there’s so many other factors that might impact how effective treatment.” (P04, Administrator)* *“I think what happens is [clinicians] really feel that [PROMs are] more for a research question for some of us academic rheumatologists and practically that it doesn’t have enough clinical utility.” (P26, Physician-Researcher)*
	Stakeholder engagement and education	*“I think what I would say about PROMs is that those who know and understand and use them are really gauged, but there’s those who are particularly outside of a research context who are very much less engaged.”* *“We try to increase [PROMs] uptake, because we’re collecting at that patient level, we want the provider to be engaged and be able to use that PROM to guide clinical based decision making equally, we want them to complete that data with that patient so that, from a program evaluation level, we can look and see is that making a difference.” (P07, Administrator)* *“I feel the same way about PREMs too, that there remains this gap between where you and I and people who are invested in [PROMs] work, where we understand and see utility for these measures and where clinicians and policymakers see utility.” (P14, Evaluator)*
Process	Balancing bottom-up and top-down approaches	*“I think sometimes we overstate the bottom-up piece … You have to create the conditions … It’s not autocratic, but it is not necessarily just where and how I might want to do this in this particular space … I do think that there is a little bit of a sweet spot.” (P02, Administrator)* *“I think that the composition of the people that are at the top layer is very different from the composition of people at the bottom, I mean, sometimes they have some commonalities, but it’s very different, the context is super different. In my humble opinion, if we have to start somewhere, doing [PROMs] at the level of engaging patients in understanding the measures and using the measures in their care, engaging the providers and doing the same, and bringing them together and understanding that, I think that will eventually infiltrate through the top, and if you see some of the international examples that been somehow the case.” (P11, Researcher)* *“And I also think the change has to come at the grassroots, like at the service delivery level, because if it comes from up top, there’s going to be pushback, [PROMs are] going to be thought of as punitive. And so you got to build it from kind of like both the top and bottom. But I think without [PROMs implementation] being established at the grassroots level, there’s not going to be any kind of desire or push from higher levels to implement these things because they’re going to see as, ‘this is just another reporting requirement to make my job harder.’ … I think it’s like a delicate balance with that.” (P21, Evaluator)*
	Patient engagement	*“I think if you can get, reach into the hearts and minds of patients, they’re the ones that are going to start asking about it.” (P15, Administrator)* *“The patient doesn’t think of [PROMs] as data, the patient thinks of it as their journey. To them, this is their symptom journey, this is those pieces of their journey that have been difficult or better or getting better, and when they look at it, they explain it differently. Patients see it as a communication tool and we’ve also had our clinicians tell us the same thing.” (P08, Administrator)* *“I think with cancer patients and in the cancer system, we have a unique opportunity in many ways, of patients that are really, really invested in sharing, talking about, looking for support through this journey. Generally, we have an engaged population that really are invested in their own care, looking for everything that is going to give them the best opportunity for positive clinical outcomes.” (P02, Administrator)*
	Specialties and Variation	*“Building those relationships needs to be done in each specialty in its own way … All the specialties are special … and they see different things being of different benefit.” (P03, Researcher)* *“I think in the sophistication of how we think about PROMs and using PROMs as part of routine care, we have to actually recognise there are different business, there are different use cases.” (P08, Administrator)* *“[PROMs implementation] depends on the condition…. You will see variations across specialities about this. That’s why I feel that one size might not always fit all. If we start tying [payment models] into outcomes, it will be tricky, because it depends on the specialty.” (P18, Researcher)*
	Workflow integration	*“Having it be effective also means that it’s low burden on the patient and the provider is automatically stored. No one has to clean up that data and then can be pulled out of a database as well. I think leveraging technology that certainly has made at least the administration and then subsequent analysis makes this a lot more seamless because prior to that, even I remember starting here, and I inherited a whole drawer full of paper, information that hadn’t been put into a database and it just doesn’t allow that information to be used or to mobilize the knowledge.” (P07, Administrator)* *“Integration with EPIC that they have created is probably one of the most novel, unique things … And because we have the ability to do some of [display function] work on our own is a real strength of the program, that will continue to allow us to be at the front edge of the implementation work. I think that these EMR changes have a huge impact on how the program will function. And if the program functions better, there’s more opportunity for it to impact on the patients.” (P01, Physician)* *“[PROMs] don’t have as much practical value at this time. I think the major issue is there’s too many isolated siloed places that we store the data and they don’t get moved into the EMR.” (P26, Physician-Researcher)* *“I would say that although that wasn’t our primary focus on how we’ve integrated the tool, the primary focus has always been on this interaction between you and your patient and what’s happened before and what you need to do for them. But now that we’ve got the Connect Care system and we’ve got the visibility of all of the dashboards and things that we’ve done, we’ve also put on our PROM directly asking patients if they want a referral.” (P08, Administrator)*
	Continuous evaluation and quality improvement	*“The impact [of PROMs at] that meso level, it’s definitely drawn attention to improving the culture of quality improvement and looking at this level of measure … We still think we’re sitting on a pile of data, that’s a goldmine that really hasn’t been accessed and utilized by all of our zone operation partners have been engaged in it.” (P09, Administrator)* *“We always talk to the clinicians and clinical leaders who are embarking on [PROMs implementation] are just using the EQ-5D as part of what we call a wellness conversation. So, I think their reaction to using or not using it, kind of helps reflect on how client self-directed they are in some ways, because if they’re saying ‘these questions are too broad, we are not able to address some of the larger picture areas.’ I think it does then speak to them what they are addressing and how they are defining clients and their goals. So, I think it can be really supportive with those conversations.” (P04, Administrator)* *“Obviously I would start to reflect on my own scores and my patients. And what am I not doing, perhaps that the others are? And maybe there’s some mutual learning there, not in a punitive sense, of course, but that continuous quality improvement cycle.” (P24, Evaluator)*

### Intervention characteristics

Stakeholders emphasized the critical need for a shared understanding of PROMs across healthcare providers, underscoring the importance of recognizing both generic and disease-specific PROMs and effectively integrating them into clinical workflows. While generic PROMs are useful, many stakeholders expressed concerns about their limitations in fully capturing the diverse and specific experiences of patients, particularly those with particular conditions. As such, there was strong support for the use of disease-specific PROMs that tailored to meet the unique needs of different patient groups. Additionally, there was a clear consensus on the need for standardization in the use of PROMs to ensure reliable data collection and integration into routine clinical practices across various settings.

Stakeholders also highlighted the advantages of using electronic systems for collecting PROMs data. Electronic methods were seen as crucial for seamless integration into existing clinical workflows, improving data storage, and enhancing overall efficiency. The importance of making PROMs data actionable at the patient-clinician level was a key point, with stakeholders stressing that simply collecting PROMs data is insufficient. For PROMs to have a meaningful impact on patient care, clear guidance and practical resources are needed to help clinicians use the data to inform clinical decisions and foster shared decision-making with patients. Finally, stakeholders acknowledged the complexity of interpreting PROMs data, emphasizing the need for context-specific training to equip clinicians with the skills to apply the data effectively and ensure its utility in clinical decision-making.

### Inner Setting

Stakeholders identified a significant need for a cultural shift within the healthcare system to align outcome measurement practices with patient-centered care principles. This shift involves moving beyond a focus on process indicators to include outcomes that reflect patients’ experiences and priorities, engaging patients, caregivers, and advocacy groups in the process. A key factor in successful PROMs implementation is changing clinicians’ mindsets and practices. Stakeholders emphasized the importance of structured change management processes to support clinicians in adopting PROMs and fostering a more patient-centered approach. Effective engagement through training, ongoing support, and clear communication about the uses of PROMs was seen as crucial for ensuring integration into clinical workflows.

Organizational commitment was also highlighted as vital for PROMs adoption. Stakeholders stressed the need for strong leadership, resource allocation, and strategic alignment with organizational goals to foster a supportive environment for PROMs use. Clear goals, dedicated infrastructure, and continuous clinician engagement were seen as necessary to sustain PROMs integration. Moreover, stakeholders advocated for embedding PROMs into broader outcome measurement frameworks, such as the triple aim or quadruple aim, to ensure their relevance and consistency across clinical settings.

Access to and integration of PROMs data were also key themes. Stakeholders highlighted the importance of user-friendly platforms that allow easy access for patients, clinicians, and administrators, enabling efficient utilization of data at various levels of the healthcare system. There was also recognition of the potential for successful PROMs implementation in specific clinical settings to serve as models for broader adoption, with local champions playing a critical role in driving these initiatives.

Finally, stakeholders stressed the importance of incentives and requirements to support PROMs adoption. The absence of such incentives and mandates was viewed as a barrier to widespread integration. Financial incentives, recognition programs, and performance-based incentives tied to PROMs utilization were suggested as possible effective motivators. Additionally, establishing formal requirements for PROMs integration could standardize practices across the healthcare system and promote a shift toward a more patient-centered culture.

### Outer setting

Stakeholders identified resource constraints as a key barrier to the successful implementation of PROMs. Limited financial and human resources hinder efforts to establish the necessary infrastructure, provide adequate training, and manage data effectively. Addressing these resource gaps is essential for facilitating the widespread adoption and effective use of PROMs across healthcare settings.

Policy and systemic challenges also emerged as critical obstacles. Stakeholders noted the complexities in policy enforcement, political influences, and the fragmentation of data systems within Alberta’s healthcare system. These systemic issues, including the lack of interoperability between platforms, made it difficult to implement PROMs consistently. There was a strong call for clear guidelines, standardized protocols, and supportive policies to streamline the adoption process and ensure greater consistency across healthcare settings.

In addition, stakeholders pointed to the limited emphasis on value-based care within Alberta’s healthcare system, which restricts the full potential of PROMs. While PROMs have significant potential to capture patient-reported outcomes that align with value-based care principles—such as focusing on patient outcomes and experiences—there is currently insufficient integration of these models into the system. The promotion of value-based care and outcome-based payment models was seen as a key area where PROMs could contribute to improving healthcare delivery.

Lastly, stakeholders stressed the importance of the ethical use of PROMs data, with a particular emphasis on maintaining privacy, confidentiality, and compliance with regulatory guidelines. Ensuring transparency and accountability in the handling of PROMs data was viewed as critical for maintaining public trust and promoting responsible data use. Without clear protocols and adherence to ethical standards, the full potential of PROMs in healthcare systems may be undermined.

### Characteristics of individuals

Stakeholders identified a significant gap in expertise and understanding of PROMs among clinicians and administrators. They emphasized the need for targeted education and training to ensure all stakeholders are equipped to select, use, and interpret PROMs effectively.

Engaging and educating patients, clinicians, and policymakers was also deemed crucial. Stakeholders highlighted the importance of empowering patients with knowledge about their PROMs data, while providing clinicians and administrators with the necessary training to incorporate this data into practice. Educating policymakers was also seen as key to fostering evidence-informed decision-making and promoting the value of PROMs in healthcare.

### Process

Successful PROMs implementation requires balancing both bottom-up and top-down approaches. Stakeholders emphasized the importance of grassroots engagement to foster ownership, coupled with top-down support to provide necessary resources and guidance. Integrating PROMs into existing clinical workflows was also seen as essential for consistent use, with stakeholders highlighting the value of embedding them into routine practices, such as through integration with electronic medical records, to ensure sustainable adoption.

Additionally, actively involving patients in the development and implementation of PROMs programs was seen as critical for enhancing data relevance and promoting wider adoption. Stakeholders advocated for incorporating patient perspectives in measure selection and working with advocacy groups to ensure measures reflect patient needs. Continuous improvement was also a key theme, with stakeholders stressing the need for regular assessments, feedback collection, and adjustments to refine the implementation process and ensure alignment with broader healthcare system goals.

### Strategies for enhancing PROMs adoption and implementation

Stakeholders highlighted several strategies to improve the adoption and utilization of PROMs across the healthcare system. The analysis of these strategies, using the Diffusion of Innovation (DOI) framework, provides a structured approach to understanding and addressing the challenges of integrating PROMs into complex healthcare settings [29]. Based on the DOI framework, the following strategies can be considered:

- *Identify and engage early adopters*: Early adopters are often influential individuals or organizations within the healthcare system. By identifying these stakeholders and involving them in the implementation process, their positive experiences and testimonies can encourage others to adopt PROMs. Providing them with the necessary support, resources, and recognition can strengthen their commitment and motivation.
- *Tailor PROMs to fit contexts*: Adapting PROMs to align with existing healthcare practices is essential for successful integration. This includes customizing PROMs to specific clinical specialties or patient populations and ensuring they are compatible with electronic medical records. Customizing PROMs for different clinical specialties or patient populations can also increase their relevance and acceptance.
- *Develop effective communication strategies*: Clear and targeted communication is key to promoting PROMs. Utilize diverse channels such as professional networks, conferences, and media to disseminate information about the benefits of PROMs. Engaging opinion leaders and sharing evidence-based success stories can address concerns, highlight the value of PROMs, and increase awareness among clinicians and administrators.
- *Establish a supportive organizational culture*: Cultivating an environment that supports PROMs is critical for their successful adoption. Leadership should emphasize the importance of PROMs in improving patient outcomes and allocate resources for training and infrastructure. Creating forums for clinicians to share experiences and best practices can foster collaboration and reinforce a culture that values PROMs.
- *Address barriers and concerns*: Identifying and mitigating barriers to PROMs adoption is essential. Common concerns include increased workload, time constraints, and skepticism about PROMs’ value. Providing targeted education and training to dispel misconceptions, along with showcasing successful case studies from similar settings, can build confidence and address these barriers effectively.
- *Provide incentives and support*: Incentives can play a significant role in encouraging the uptake of PROMs. Financial incentives, recognition programs, and professional development opportunities can motivate clinicians and organizations to prioritize PROMs. Additionally, offering ongoing technical support, including training, data analysis tools, and interpretation guidance, can facilitate the practical use of PROMs in clinical decision-making.
- *Monitor and evaluate the impact*: Continuous monitoring and evaluation are vital to demonstrate the value of PROMs. It is critical to collect and analyze data on patient outcomes, clinician satisfaction, and improvements in healthcare quality to evaluate the impact of using PROMs in a given clinical setting. Sharing these results with stakeholders can highlight the benefits of PROMs and encourage further adoption.

## Discussion

Significant progress has been made in implementing PROMs within the Alberta healthcare system, with positive engagement observed from certain leaders and clinical areas. However, for PROMs to reach their full potential, comprehensive whole-system adoption and integration are essential. While key stakeholders have recognized the value of PROMs, their adoption has not been universal throughout the healthcare system. Achieving widespread adoption requires a common vision and engagement across various sectors, specialties, and administrative levels. Overcoming barriers like limited resources, lack of standardized processes, and resistance to change is crucial. Collaboration among healthcare leaders, researchers, policymakers, and patients is vital to drive whole-system adoption, facilitating the sharing of experiences, best practices, and lessons learned.

Stakeholders highlighted the importance of transforming the healthcare culture to prioritize patient-centered care and value-driven outcomes measurement, including integrating PROMs as a crucial component. They acknowledged challenges and opportunities in implementing PROMs to drive meaningful change in the health system, emphasizing the need for a clear vision and supportive leadership. Raising awareness and building capacity in using PROMs and reporting data were identified as essential steps. Patient engagement was recognized as a catalyst for PROMs adoption. To maximize the impact of PROMs data, establishing a robust feedback loop is crucial, integrating insights into clinical practice and quality improvement. Ongoing evaluation, monitoring, and dissemination of PROMs data are necessary to empower healthcare providers with actionable information for improved patient outcomes.

Our findings align with previous initiatives and studies that have examined the factors influencing the implementation and utilization of PROMs in health systems, some of which have also utilized the CFIR framework. Stover and colleagues identified consistent barriers to PROMs implementation, including technology-related challenges, uncertainty about their appropriate utilization, and competing demands from established clinical workflows across different patient populations and care settings. Enablers were found to be context-specific, highlighting the importance of tailored implementation strategies based on clinic resources [30]. Hyland and colleagues identified facilitators and barriers within each domain of the CFIR framework for successful implementation [31]. Key facilitators included allowing clinicians to select PROM measures and ensuring a user-friendly data platform (intervention), adapting data collection to patient home environments (outer setting), informing clinicians about the multifaceted use of PROM data (inner setting), integrating PROM education into clinician training (characteristics of individuals), and establishing specialty-agnostic PROM implementation teams (process) [31]. Van Oers and colleagues evaluated the implementation process of the KLIK PROM program in the Netherlands using CFIR and found that successful implementation was influenced by intervention characteristics (such as ease of use), characteristics of individuals (such as motivation), and the implementation process (including support) [32]. They identified thirteen CFIR constructs as current barriers to implementing the KLIK PROM portal and recommended identifying and preparing champions as the highest recommended implementation strategy to address these barriers.

A recent systematic review that aimed to identify factors that influence the implementation of electronic PROMs in healthcare settings, and included 24 studies, identified 96 categories that were mapped across all CFIR domains [33]. The review highlighted that point-of-care utilization of PROMs could strengthen the patient’s voice through improved communication and a focus on shared decision-making in the patient’s healthcare journey. However, it also identified that the integration of PROMs into existing electronic medical records and clinical workflows remains a key challenge. These two key themes align with those identified in our exploration.

Recognizing the distinctive characteristics of Alberta’s healthcare system, along with identifying the contextual factors affecting PROMs implementation, allows us to develop targeted strategies to leverage PROMs data for meaningful improvements in patient care. Achieving this requires close collaboration among healthcare providers, administrators, researchers, and policymakers to set clear goals for measuring PROMs within Alberta’s health system, define key performance indicators—including patient-reported outcomes—and implement data-driven interventions. A crucial aspect of this process is the seamless integration of PROMs data into existing electronic medical record systems, enabling efficient data capture, storage, analysis, and reporting to all stakeholders, including patients and clinicians.

This stakeholder engagement and consultation initiative is planned as an ongoing process that will involve gathering patients’ perspectives and experiences with PROMs at later stages.

Mapping the factors influencing the implementation of PROMs to the CFIR and DOI frameworks offered a structured method for analyzing the dynamics of adoption. This comprehensive approach will enable designing strategies to enhance the adoption, implementation and utilization of PROMs data across the whole healthcare system, ultimately improving healthcare delivery and patient experiences throughout the province.

### Strengths and Limitations

A key strength of this study lies in its purposive sampling approach, which ensured a diverse range of perspectives from stakeholders across various sectors of the Alberta healthcare system, including clinicians, administrators, and decision-makers. This broad representation, alongside recommendations from participants for additional key stakeholders, provided a comprehensive view of the factors influencing PROMs adoption and use. The use of well-established frameworks like the CFIR and DOI, along with a robust thematic analysis, enhanced the rigor and comparability of the findings with other regional studies. Additionally, the iterative process of data collection and validation through stakeholder feedback at the town hall discussion helped increase the credibility of the results.

However, there are several limitations to this study. The involvement of existing collaborators and prior working relationships with some participants may have introduced potential biases, as these connections could have influenced the direction or depth of the discussions. Additionally, while the study aimed to capture a wide range of perspectives, certain groups—particularly those outside primary healthcare or administrative sectors—may still be underrepresented. Notably, one key perspective missing from this project is that of patients themselves. Although the initial phase of the stakeholder consultation was focused on other key groups, patient engagement was planned for a subsequent phase. This was due to the fact that our team was in the process of establishing a patient engagement network, which was not fully operational at the time of this study. Therefore, patient input was deferred, but will be a critical component of future consultation activities.

## Conclusion

This study identified factors impacting the adoption and implementation of PROMs in Alberta’s healthcare system, and key strategies to enhance their use. These included fostering a unified understanding of PROMs, integrating them into clinical workflows, and leveraging electronic data collection. Emphasizing patient-centered care, securing organizational commitment, and addressing resource constraints and policy challenges are critical. Additionally, targeted education for stakeholders and continuous evaluation of PROMs impact are essential. Implementing these strategies, including engaging early adopters, tailoring PROMs to contexts, and providing incentives, can significantly improve PROMs integration and ultimately enhance patient care outcomes.

## Electronic supplementary material

Below is the link to the electronic supplementary material.

Supplementary Material 1

## Data Availability

The data, including interview transcripts, will not be shared. Stakeholders were informed that their input and insights would be analyzed and reported in an aggregated form, ensuring their individual responses remain confidential.

## Abbreviations

PROMs: Patient-reported outcome measures
CFIR: Consolidated framework of implementation research
DOI: Diffusion of innovation
AHS: Alberta Health Services
