# Supplementary material for: Stakeholders’ perspectives on implementing and integrating patient-reported outcome measures (PROMs) in health systems - insights from Alberta, Canada
Source: J Patient Rep Outcomes. 2025 Jun 5;9:63. doi: 10.1186/s41687-025-00887-0 (PMC12141181; doi:10.1186/s41687-025-00887-0)
Supplement: Supplementary file 1 — Supplementary Material 1 [file 41687_2025_887_MOESM1_ESM.pdf]

Interview Guide

Introduction:

- Explain the purpose of the interview and the importance of their insights.
- Obtain their verbal informed consent for the interview and its recording.

Participant name:
Participant ID: _______________ **Interview date: ______________**

Location: ____________ (city)

What is your current position/affiliation? ______________/________________

What are your current roles (check all that apply)?

Clinician
Researcher
Academic
Administrator
Policy maker
Other: ________

As a key stakeholder within the Alberta healthcare system, we would like to gain your insights on the use of patient-reported outcome measures (PROMs). Alberta has been investing in the implementation of PROMs for several years, with varying levels of success across different settings. The collection of routine PROMs data presents a significant opportunity for healthcare decision-making, and we would like to explore your thoughts on the best ways to utilize this information. We would like to ask you about your opinion on how we can use PROMs data here in the province, and identify factors that can help us in enhancing such use.

Today, we want to talk about the use of aggregate PROMs data at the micro, meso and macro levels within the system. Here, I'd like to share with you my screen to show you the framework that I am referring to [SCREEN SHARE].

If the participant works in specific clinical area, add "in your clinical setting" to the question

Question	Prompt
To start, can you share your insights on the implementation and use of PROMs within the Alberta healthcare system? What has been your experience with PROMs so far and what are your thoughts on its impact in the province?	How do you perceive the uptake of PROMs across different healthcare settings? Are there any specific examples or challenges you can share that highlight the impact of PROMs on patient care and healthcare delivery in Alberta?
From your perspective, what are the most effective ways to utilize routinely collected PROMs data in the context of healthcare service planning and evaluation? How can PROMs data be leveraged to inform decision-making	In terms of healthcare service planning, how should PROMs data be incorporated into existing structures or processes? Can you identify any specific decision-making areas where PROMs data could improve outcomes or efficiency?

and enhance service delivery in Alberta?	Facilitators and barriers: What factors help or hinder the integration of PROMs data into decision-making processes?
In your view, what role can routinely collected PROMs data play in quality improvement initiatives and performance evaluation within the Alberta healthcare system? How can PROMs data be leveraged to compare the performance of different providers, clinics, service units, or hospitals?	What are the key quality improvement areas where PROMs data could have the most impact?<br>How can PROMs data help to highlight areas of excellence or opportunities for improvement within healthcare institutions?<br>Facilitators and barriers: What challenges or obstacles might arise when using PROMs for performance evaluation?
From your experience, how can routinely collected PROMs data inform and shape provincial health priorities in Alberta? In your opinion, what is the potential impact of PROMs data on decision-making and policy development at the provincial level?	How can PROMs data be integrated into provincial health policy development?<br>What types of provincial health priorities could be influenced by PROMs data, and why?<br>Facilitators and barriers: What factors may help or hinder the use of PROMs data in shaping policy decisions?
In your view, what role can routinely collected PROMs data play in outcome-based payment models like value-based care? How can PROMs data be leveraged to support the shift towards a value-based healthcare system in Alberta?	How can PROMs data be used to assess the effectiveness of healthcare interventions and inform payment structures?<br>What are the potential challenges and opportunities in implementing value-based care using PROMs data?
In your opinion, would making routinely collected PROMs data publicly available support informed decision-making for patients when choosing their healthcare providers? How can PROMs data be made accessible in a responsible and meaningful way to support patient choice?	What are the potential advantages and risks of making PROMs data public?<br>How can healthcare providers ensure that PROMs data is presented in a way that is accessible and useful to patients without overwhelming them?<br>Facilitators and barriers: What might prevent patients from fully utilizing publicly available PROMs data?
From your experience, what steps or initiatives are necessary to fully realize the potential of routinely collected PROMs data in Alberta's healthcare system? In your view, what challenges need to be addressed, and what solutions do you propose to enhance the utilization of PROMs data?	What types of financial, operational, human, and technical supports are needed to optimize the use of PROMs data?<br>Are there any organizational or system-level changes that could better support the effective use of PROMs data?<br>Facilitators and barriers: What specific barriers do you see in terms of infrastructure, training, or policy that may limit the widespread use of PROMs data?
Anything else you'd like to add before we conclude the interview?	Is there any additional insight or recommendation you'd like to share regarding the implementation, challenges, or future of PROMs in Alberta's healthcare system?<br>Are there any emerging trends or innovations in healthcare that you believe could further impact the use of PROMs?

Is there anyone else that you think we should talk to?

Thank you for taking the time to complete this interview and share you perspectives with us. Your insights are very valuable.
